# Effect of Annealing on Vacancy-Type Defects and Heterogeneous Cu Precipitation Behavior in Fe_60_Cr_12_Mn_8_Cu_15_Mo_3_V_2_ Alloy

**DOI:** 10.3390/ma18112613

**Published:** 2025-06-03

**Authors:** Fengjiao Ye, Te Zhu, Peng Zhang, Peng Kuang, Haibiao Wu, Xingzhong Cao

**Affiliations:** 1 Institute for High Energy Physics, Beijing 100039, China; yefj2018@163.com (F.Y.); zhute@ihep.ac.cn (T.Z.); zhangpeng@ihep.ac.cn (P.Z.); kuangp@ihep.ac.cn (P.K.); 2 School of Nuclear Science and Technology, University of South China, Hengyang 421001, China; 3 The First Affilated Hospital of University of South China, Hengyang 421001, China

**Keywords:** Fe_60_Cr_12_Mn_8_Cu_15_Mo_3_V_2_ alloy, annealing, vacancy-type defects, Cu nanoprecipitates

## Abstract

This study systematically investigates the evolution of vacancy-type defects and heterogeneous Cu nanoprecipitates in an Fe_60_Cr_12_Mn_8_Cu_15_Mo_3_V_2_ (at%) multi-principal element alloy during thermal processing, utilizing Positron annihilation lifetime spectroscopy (PAS), coincidence Doppler broadening (CDB) spectroscopy, and transmission electron microscopy (TEM). The results show that the alloy exhibited a dual-phase coexistence structure of Body-Centered Cubic (BCC) and Face-Centered Cubic (FCC). The CDB results show that the density of heterogeneous Cu precipitates gradually increases with annealing temperature. Compared to the as-cast alloy, the precipitates annealed at 773 K exhibit a significantly reduced size (approximately 33 nm) with higher density. The PAS results demonstrate that gradual migration and aggregation of monovacancies at 573 K form vacancy clusters, while contraction and dissociation of these clusters dominate at 673 K. Within the temperature range of 773–973 K, the dynamic equilibrium between the aggregation and decomposition of vacancy clusters maintains stable annihilation characteristics with minimal lifetime changes.

## 1. Introduction

The current capabilities of structural materials in withstanding harsh environments exhibit inherent limitations, rendering conventional alloys insufficient to meet the stringent design demands of nuclear reactors [1,2,3,4]. The development and screening of nuclear materials constitute a rigorous and extensive process that necessitates a dual-pronged approach [5,6,7,8,9]. Firstly, it is imperative to modify existing nuclear materials to adapt to new requirements based on anticipated future service environment changes. Secondly, there is an urgent need to develop novel materials with potential applications in nuclear energy systems [10,11,12,13]. Currently, two fundamental approaches are employed to enhance the irradiation resistance of nuclear materials: one involves incorporating nano-sized precipitates or other defect traps within the material to “accommodate” irradiation-induced defects [14,15,16,17,18], thereby facilitating rapid recovery of these defects; the other involves exploring novel materials with higher defect formation or migration energies [19,20,21,22,23]. Multi-component alloys represent a breakthrough in traditional alloy design concepts. Their chemical complexity, high-entropy effects, severe lattice distortions, and sluggish diffusion collectively contribute to their exceptional physical properties. In recent years, research on alloys has expanded across various disciplines, leading researchers to recognize that alloys synthesized with different elemental ratios tend to form non-single-phase solid solutions and are prone to forming solute atom clusters or even precipitates. Interestingly, numerous studies have shown that pre-introduced precipitate phases can significantly enhance the physical properties of alloys by affecting strain-hardening mechanisms and acting as defect traps to capture radiation-induced defects, thereby improving radiation resistance [24,25,26,27]. For example, the addition of Ti (N, C, O), Y_2_Ti_2_O_7_, Y_2_TiO_5_, and Y-Ti-O nanoclusters to oxide dispersion-strengthened (ODS) alloys improves both the high-temperature strength and radiation resistance of these alloys [28,29,30]. Hadraba et al. introduced Y_2_O_3_ nanoparticles into FeCoMnCrNi alloys, resulting in obvious grain refinement and notable improvements in yield strength at both room and elevated temperatures [31]. Building on this concept, adding elements that can produce precipitated phases to alloys is expected to adjust tensile strength, plasticity, and radiation resistance. By carefully adjusting the alloy components and ratios, an Fe_60_Cr_12_Mn_8_Cu_15_Mo_3_V_2_ (at%) alloy without high-activating elements was prepared in this study. In this alloy, an Fe content of 60 at% ensures that the alloy retains the cost effectiveness and machinability inherent to an Fe-based alloy, while performance optimization is achieved through the addition of other elements. Chromium (Cr) provides resistance to oxidation and corrosion in acidic environments. The 8 at% Mn content enhances wear resistance. The high Cu content (15 at%) facilitates the formation of Cu-rich phases during heat treatment. Vanadium (V) at 2 at% contributes to improving the plasticity of the alloy. The Cu content in Fe_60_Cr_12_Mn_8_Cu_15_Mo_3_V_2_ is only 15%, which is not considered highly saturated. Although molybdenum (Mo) is a highly reactive element, a small amount (3 at%) is incorporated to enhance the alloy’s strength.

Irradiation-induced Cu precipitation has been identified as a major factor in the embrittlement of reactor pressure vessel steel under ion irradiation, particularly when Cu content is highly supersaturated [22,23,24,25,26,27,28,29,30,31,32,33,34]. However, irradiation embrittlement still occurs in RPV steel even without Cu addition, indicating that embrittlement cannot be solely attributed to CRPs. Additionally, the yield strength and ductility of high-strength low-carbon steel improve due to CRP precipitation. Adjusting the alloy composition is an effective method to reduce radiation embrittlement and enhance resistance in many commercial nuclear alloys. For instance, high-density nanoprecipitation can serve as absorption sinks for vacancies and defects, thus becoming composite centers [35]. Although many studies focus on irradiation-induced nanoprecipitate formation in RPV steel, fewer studies examine the evolution of existing nanoprecipitates during irradiation and their impact on irradiation resistance. Some studies have shown that after high-dose irradiation, alloy steels containing CRPs do not exhibit radiation hardening or embrittlement but instead demonstrate radiation softening, ensuring better service performance and safety. In this work, CRPs are pre-introduced rather than generated by irradiation. Therefore, the pre-engineered Cu nanoparticles in this study are different from irradiation-induced precipitation, which helps to improve the irradiation embrittlement resistance of the alloy, which has been reported in previous works [36]. This study investigates the migration and recovery of micro-defects in the alloy under various heat treatment conditions, as well as the characteristic changes in heterostructured Cu nanoparticles.

## 2. Materials and Methods

### 2.1. Materials

The Fe_60_Cr_12_Mn_8_Cu_15_Mo_3_V_2_ alloy was synthesized via vacuum arc melting under high-vacuum conditions (<3 × 10^−4^ Pa) using high-purity elemental constituents (Fe, Cr, Mn, Cu, Mo, V > 99.9 wt%). In this work, this alloy was named A1. Prior to melting, the chamber atmosphere was replaced with argon through multiple gas-purging cycles. To ensure chemical homogeneity, the ingot underwent six successive remelting cycles with electromagnetic stirring. The cast alloy was subsequently cut into square samples (10 × 10 × 1 m^3^), and cleaned with acetone to remove surface contaminants. These samples were ground with SiC abrasive papers of varying particle sizes followed by mechanical polishing to achieve a smooth mirror finish. Finally, surface stress was relieved through electrolytic polishing. Isothermal annealing is carried out from 373 K to 973 K at a heating rate of 10°/min, with an interval of 100 K and a holding time of 1 h.

### 2.2. Characterization

The alloy’s microstructure was analyzed using X-ray diffraction (XRD, D8 Advance, Bruker Company, Germany), with a scanning rate of 4°min^−1^, employing Jade 6.0 analysis suite for phase identification. Scanning electron microscopy (SEM, Hitachi S-4800, Hitachi Company, Japan) equipped with energy-dispersive X-ray spectroscopy was employed for microstructural and morphological characterization of the different samples, facilitating analysis of precipitated phases after heat treatment. The sample was cut into circular pieces with a diameter of 3 mm, and mechanical polishing was used to thin the 3 mm disk to a thickness of approximately 70 µm. The Gatan 691 precision ion polishing system was used to perform ion beam milling thinning on the sample under the conditions of instrument voltage of 20 V and temperature of around −25 °C, using 5% perchloric acid and 95% ethanol solution. Finally, the characteristics of Cu precipitates in the alloy were characterized by transmission electron microscopy (TEM, FEI Tecnai F20, FEI Company, USA) equipped with High-Angle Annular Dark Field (HAADF) and energy-dispersive X-ray spectroscopy (EDX, Bruker Company, USA). The defects of alloy during annealing were characterized by Positron annihilation spectroscopy (PAS, Self-developed), the time resolution was 196 ps [37], and the acquired data were analyzed through LT 9.0 software. Two identical samples had a 10 µCi ^22^Na positron source sandwiched between them, ensuring that each spectrum contained a substantial count of 10^6^. Two HPGe (high-purity Ge) detectors were used to measure the thermal stability and precipitation behavior of the Cu precipitates through coincidence Doppler broadening spectroscopy (CDB, Self-developed). CDB spectra are characterized by a high peak-to-valley ratio and have significant advantages in characterizing high-momentum core electrons, enabling the measurement of the momentum distribution of core electrons. Compared to valence electrons, the annihilation probability of core electrons and positrons in elements is relatively small, and the annihilation of core electrons produces a larger Doppler shift. Therefore, the momentum distribution information of the core electrons can be obtained from the tail region of the Doppler broadening curve [38]. CDB measurement simultaneously detects two gamma photons produced by annihilation and matches their time and energy to eliminate the influence of the background, which can separate the annihilation information of high-momentum electrons. For CDB spectral, two main regions can be distinguished: a low-momentum region near the peak position (*P*_L_ < |3 × 10^−3^ m_0_c|), reflecting the annihilation of valence electrons; and a high-momentum region (|6.2 × 10^−3^ m_0_c| < *P*_L_), which is related to the annihilation of high-momentum electrons [39]. Therefore, the signal of positron core electron annihilation can be used to identify the characteristic information of Cu atoms. PAS and CDB measurements were carried out at the Institute of High Energy Physics.

## 3. Research Results and Their Analysis

### 3.1. The Influence of Annealing on the Microstructure of Alloys

**Figure 1:**
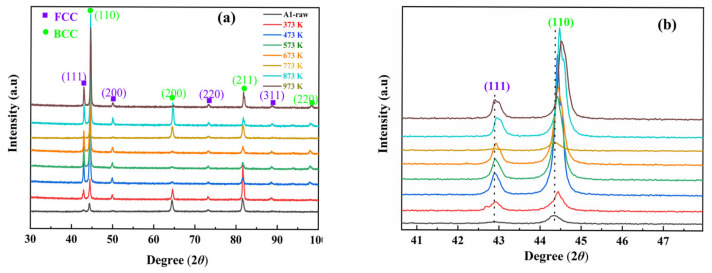
(**a**) XRD diagram of the alloy after annealing treatment at 373 K~973 K; (**b**) enlarged diagrams of main diffraction peaks after annealing treatment.

Figure 1a displays the XRD patterns of the alloy across annealing temperatures. The as-cast alloy exhibits a dual-phase structure comprising BCC (72.4 wt%) and FCC (27.6 wt%) phases, as quantified through Jade 6 software Rietveld refinement. The FCC phase formation correlates with Cu precipitation. The lattice constants of BCC and FCC structures in the alloy are measured to be 2.877 and 3.662 Å, respectively [40]. Notably, no secondary phases emerged in the XRD patterns even after annealing up to 973 K. Compared to the as-cast alloy, the intensity of main diffraction peaks gradually increased with increasing annealing temperature. Furthermore, as illustrated in Figure 1b, the diffraction peaks of BCC (110) and FCC (111) shifted to higher angles with increasing annealing temperature. This shift is attributed to changes in crystallization, grain size, and lattice distortion caused by high-temperature heat treatment.

**Figure 2:**
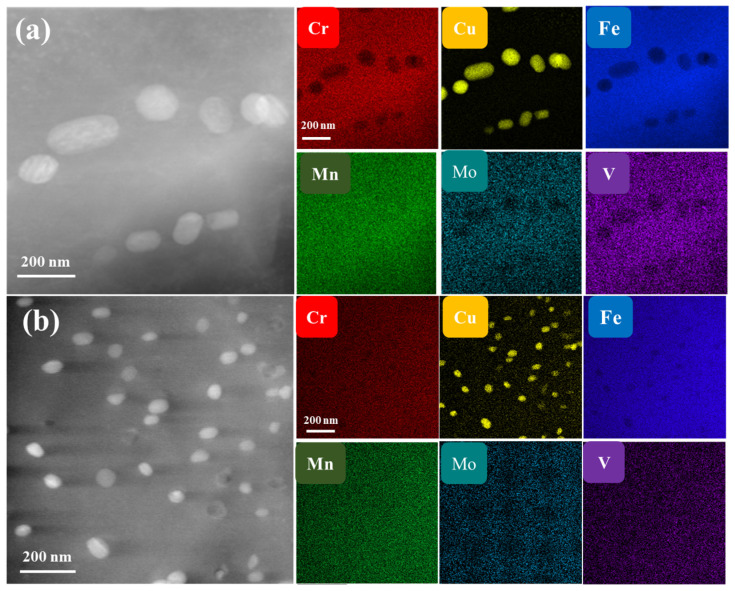
HAADF-STEM results and corresponding elemental maps of the alloy (**a**) as cast [37], (**b**) after annealing at 773 K for 1 h.

The morphology of as-cast samples is presented in Figure 2a [37]. It can be observed that there is a large-sized Cu precipitation, with an average size of approximately 90 nm, mainly distributed along the dislocation lines. The Cr, Fe, Mo, V Elements are removed from the precipitates, but uniformly distributed throughout the alloy. After annealing at 773 K, as can be seen in Figure 2b, the size of precipitated phase decreases significantly, and spherical and elliptic precipitates are present both in the as-cast sample and in the annealed alloy with a random distribution.

**Figure 3:**
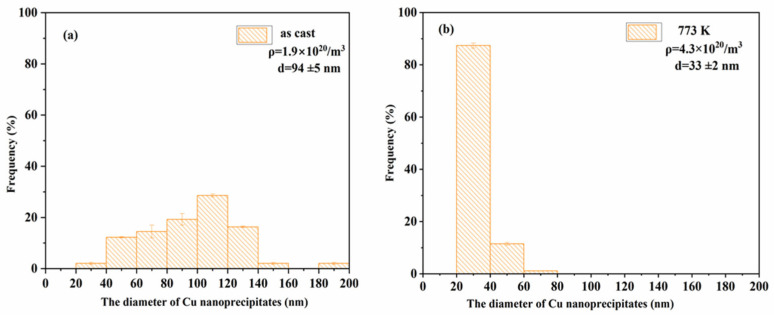
Size distribution of Cu nanoprecipitates for different samples—(**a**) as cast, (**b**) after annealing at 773 K for 1 h.

To systematically investigate annealing temperature effects on Cu precipitate characteristics, statistically representative populations (>100 precipitates per condition) were quantitatively analyzed from TEM micrographs of as-cast specimens and those annealed at 773 K. This comparative approach enables precise evaluation of thermal treatment impacts on precipitation size distribution and number density evolution. Figure 3 reveals significant annealing-induced modifications in Cu precipitation characteristics. Quantitative TEM analysis demonstrates the average precipitate size from 94 nm (as-cast) to 33 nm following 773 K, accompanied by a twofold increase in number density to 4.3 × 10^20^ m^−3^. This inverse correlation between precipitate size and population density aligns with subsequent PAS and CDB analyses, indicating enhanced precipitation refinement through thermal treatment.

### 3.2. Effect of Annealing on Defects and Cu Precipitation Behavior

**Figure 4:**
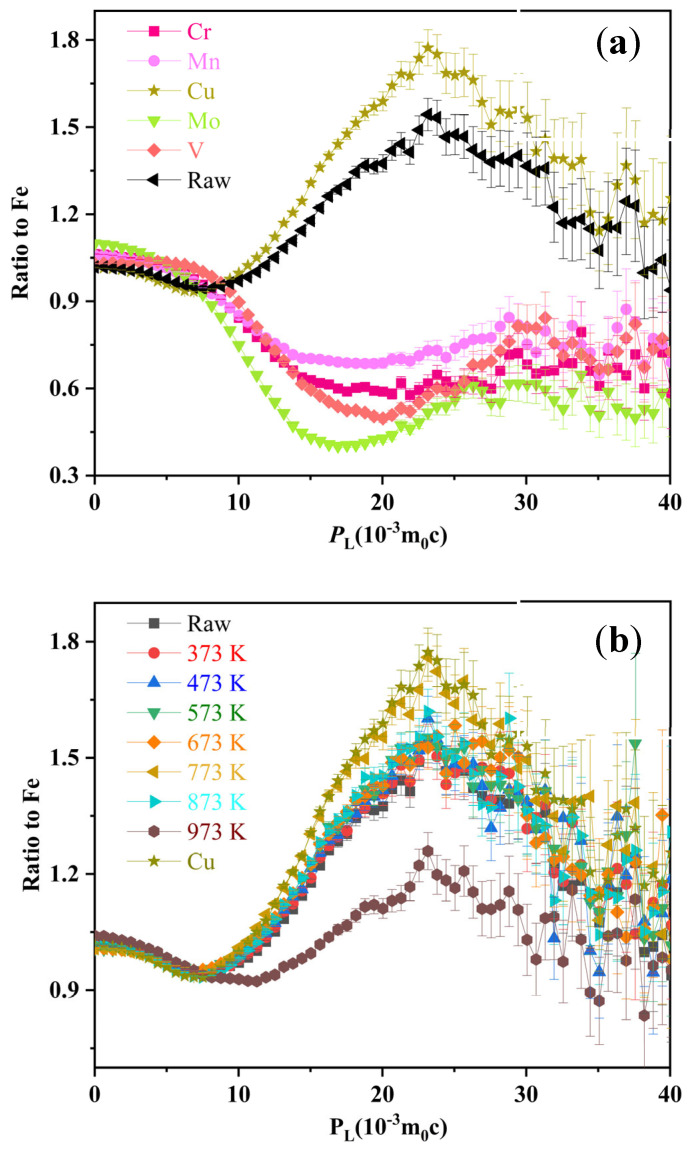
(**a**) CDB ratio curve of cast alloy and pure elements to pure Fe; (**b**) CDB ratio curve of as-cast and annealed alloys at different temperatures, pure Cu to pure Fe.

To characterize the evolution behavior of Cu nanoparticles more directly, the CDB ratio to Fe curves of alloy samples under different annealing states was obtained. The high-momentum region (|*P*_L_|> 3 × 10^−3^ m_0_c) reflects the information of positrons annihilation with core electrons [41,42,43]. The low-momentum (|*P*_L_| < 3 × 10^−3^ m_0_c) region reflects the information of vacancy-type defects [44,45]. To characterize the elemental precipitation behavior of this alloy at different temperatures, the curves of pure elements are also displayed in Figure 4a. The curve of Cu exhibits a peak near *P*_L_ = 2.25 × 10^−2^ m_0_c, while the curves of pure Cr, V, and Mo exhibit valley features at approximately *P*_L_ = 2.1 × 10^−2^ m_0_c, 2.0 × 10^−2^ m_0_c, and 1.8 × 10^−2^ m_0_c, respectively [37,45]. Pure Mn shows a shallow plateau at ~1.5 × 10^−2^ m_0_c. The CDB curve of the as-cast alloy shows a peak like that of pure Cu near *P*_L_ = 2.25 × 10^−2^ m_0_c, indicating that positrons annihilate with the 3d electrons of Cu element. The XRD results also confirm that the FCC structure in the alloy can be attributed to the heterogeneous Cu precipitation.

Figure 4b shows that the characteristic peak value of the alloy’s CDB curve increases gradually with increasing annealing temperature. The CDB curves of the as-cast samples are higher than 1 in the low-momentum region, which indicates that more positrons annihilate with vacancy. When the annealing temperature reaches 773 K, the characteristic peak of the CDB curve closely matches that of pure Cu, and the characteristic peak decreases with further increases in annealing temperature. These results indicate that as the annealing temperature increases to 773 K, the amount of small-sized Cu precipitates gradually increases. The size of precipitated phases in Fe-based solid solutions is closely related to annealing temperature and time. During low-temperature annealing (e.g., <773 K), defect-assisted nucleation and spinodal decomposition dominate, facilitating the formation of uniformly distributed small-sized Cu precipitates [46,47]. With the increase of temperature, the Cu precipitated phase undergoes dissolution and re-nucleation processes. The migration of grain boundaries at high temperatures will promote the coalescence of the precipitated phase [46,48]. Consequently, annealing temperatures exceeding 773 K may induce noticeable coarsening of Cu precipitates. Future investigations will systematically explore the temperature-dependent evolution characteristics of Cu precipitates in multi-principal element alloys, with particular emphasis on elucidating the underlying physical mechanisms governing their phase transformation kinetics and microstructural reorganization.

**Figure 5:**
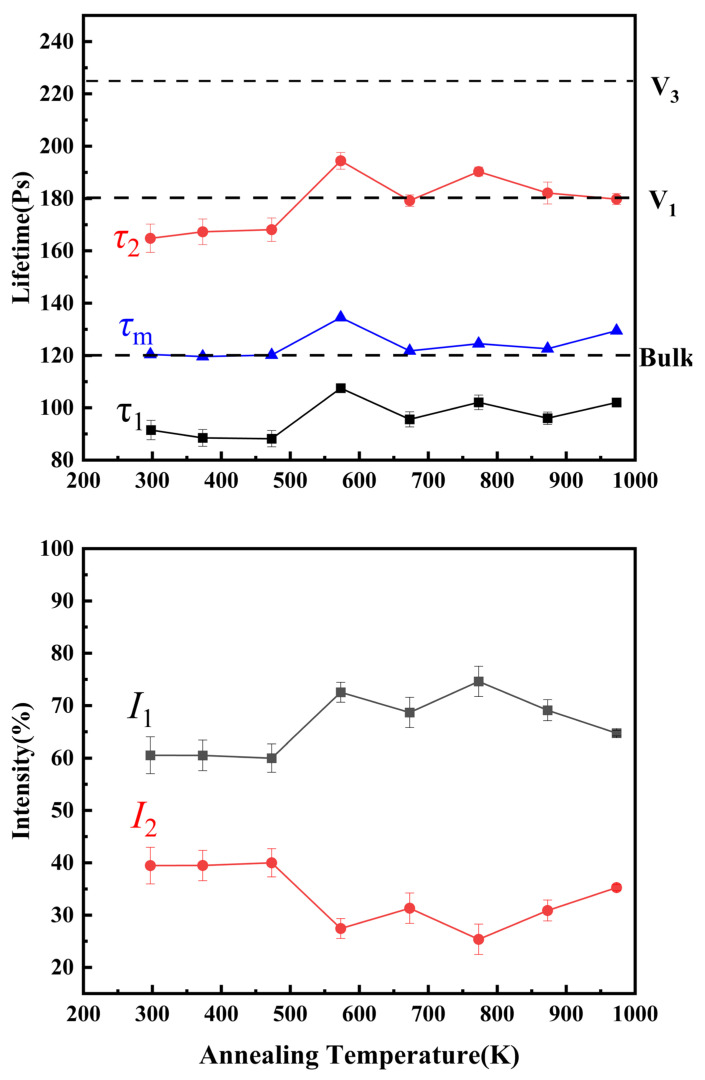
Positron annihilation lifetime spectra of the alloy annealed at various temperatures.

Figure 5 shows the positron annihilation lifetime for the alloy after annealing at various temperatures. Table S1 in the Supplementary Materials presents the positron annihilation lifetime values and corresponding intensity parameters of the annealed samples at different temperatures. Figure S1 in the Supplementary Materials shows the PAS spectra and relative fitting curves of samples annealed at 373 K. The variations in the long lifetime *τ*_2_ and the corresponding intensity *I*_2_ are attributed to the co-evolution of vacancy and Cu precipitates during the annealing process at different temperatures. The variations in lifetimes *τ*_1_, *τ*_2_ and the average positron lifetime *τ*_m_ with temperature are shown in Figure 5 [25,26,27]. *τ*_1_ represents the information of free electrons, while *τ*_2_ represents the information of positron annihilation with vacancy defects. The average positron lifetime (*τ*_m_) is derived from the weighted average of two components in the lifetime spectrum. *τ*_m_ reflects the overall defect environment in the alloy. The two-state trapping model is employed to analyze the evolution of defects and Cu nanoparticles within the alloy [49,50,51]. The as-cast alloy exhibits positron lifetimes *τ*_1_ = 91.5 ps and *τ*_2_ = 164.8 ps (intensity *I*_2_ = 39.5%), values aligning with edge vacancy–dislocation complexes reported for pure Fe [43]. These results indicate that low-density vacancies containing dislocations formed during the alloy’s melting and cooling stages due to internal stresses induced by temperature gradients between the inner and outer surfaces, serving as effective positron trapping sites (e.g., defects). When annealed at 373 K and 473 K, the long lifetime component (*τ*_2_) remained approximately 168 ps with no significant changes in lifetime or intensity values, suggesting that these lower annealing temperatures failed to reach the vacancy migration threshold. However, due to the migration temperature of vacancies in pure Fe being 423 K, and the absence of vacancy migration at the annealing temperature of 473 K in this study, this indicates that the vacancy migration temperature of the multi-component alloy is significantly higher than that of pure Fe [40,49]. The observed enhancement originates from elevated vacancy migration energy barriers, intensified lattice distortion, and multi-element synergy. As the annealing temperature increases to 573 K, the *τ*_2_ increases to 194 ps. The lifetime of vacancy clusters containing three vacancies in pure Fe is about 225 ps [35]. The theoretical calculation of positron lifetime for single vacancies is about 180 ps. Therefore, there may be double-vacancy defects in the sample when the annealing temperature is 573 K. This indicates that the energy provided by high temperature leads to the gradual migration and aggregation of single vacancies in the alloy, forming vacancy clusters. At the same time, the intensity *I*_2_ decreased to 27.4%. From 573 K to 673 K, the long lifetime component decreased from 194 ps to 179.2 ps, accompanied by increased *I*_2_ intensity. This is approximately the lifetime of the single vacancy feature (180 ps in Fe), indicating that the vacancy cluster partially dissociates into monovacancy, and the corresponding intensity *I*_2_ increases. The long lifetime *τ*_2_ of samples annealed within the temperature range of 673–973 K exhibits a characteristic trend of an initial increase followed by a subsequent decrease. Previous studies have demonstrated that in neutron-irradiated Fe-Cu alloys, Cu atoms preferentially segregate to void surfaces. The progressive accumulation of Cu precipitates induces a corresponding reduction in void dimensions [52], thereby leading to decreased lifetime values. The observed lifetime reduction at 973 K annealing temperature may be attributed to microstructural evolution of Cu precipitates. As the temperature rises, the dual competing mechanisms of Cu precipitation and vacancy dynamics collectively influence the lifetime behavior: increased Cu precipitation promotes lifetime reduction, while vacancy dissociation enhances the *τ*_2_ values. This counteractive relationship enables the reasonable inference that vacancy concentration surpasses that of Cu precipitates in the alloy system. Consequently, the variation in long lifetime within the 673–973 K thermal regime fundamentally originates from the temperature-dependent interplay between vacancy cluster evolution (aggregation and dissociation processes) and Cu precipitation kinetics.

## 4. Conclusions

An Fe_60_Cr_12_Mn_8_Cu_15_Mo_3_V_2_ alloy with low activation and Cu nanoprecipitates was fabricated by adjusting the composition and proportion of the alloy. The microstructure of the alloy was regulated at different annealing temperatures, and the effect of thermal effects on the Cu nanoprecipitates and defect was investigated. The results indicate that the as-cast alloy presents a coexistence of BCC and FCC structures. With the increase in annealing temperature, the density of heterogeneous Cu nanoparticles gradually rises, and the precipitate size is approximately 33 nm at 773 K, reaching the maximum density. After annealing at 773 K, the high density and small size heterogeneous Cu precipitation will contribute to enhancing the excellent radiation resistance of the alloy. The positron annihilation lifetime results showed that during annealing at 573 K, single vacancies migrated and generated double-vacancy clusters, while at 673 K, the spatial clusters dissociated due to thermal activation. As the annealing temperature increased to 773–973 K, the dynamic equilibrium of aggregation decomposition of vacancy clusters led to the stabilization of the annihilation lifetime parameter.

## Data Availability

The data presented in this study are available on reasonable request from the corresponding author due to privacy.

## Supplementary Materials

The following supporting information can be downloaded at: https://www.mdpi.com/article/10.3390/ma18112613/s1, Table S1: Positron annihilation parameters of samples at different annealing temperatures; Figure S1: PAS spectra and relative fitting curves of samples annealed at 373 K.

## References

[1] Moschetti M., Burr P.A., Obbard E., Kruzic J.J., Hosemann P., Gludovatz B. (2022). Design considerations for high entropy alloys in advanced nuclear applications. J. Nucl. Mater..

[2] Zhang L., Dou Y.K., Bai B., Yu B.T., He X.F., Yang W. (2024). Research progress on creep resistance of high entropy alloys. J. Alloys Compd..

[3] Rodríguez-López A., Savoini B., Monge M.A., Galatanu A., Galatanu M. (2024). Evaluation of thermal properties of CuCrFeV (Ti, Ta, W, Mo) for nuclear fusion applications. Nucl. Mater. Energy.

[4] Chu S., Majumdar A. (2012). Opportunities and challenges for a sustainable energy future. Nature.

[5] Priyanka A.D., Abhijeet D., Hu Z.H., Dubey M., Shao L., Prabhakaran R., Mishra R.S. (2024). Irradiation-induced shift in the thermodynamic stability of phases and the self-healing effect in transformative high entropy alloys. J. Nucl. Mater..

[6] Wan H., Su Z.X., Yan X., Yang J., Lu Y.P., Shi T., Lu C.Y. (2024). Microstructures and hardening effects of refractory high entropy alloys irradiated by Proton & He ion dual beam. Mater. Charact..

[7] Abid H., Dhaka R.S., Jin R.H., Kumar S.S., Kumar K.P. (2023). A critical review on temperature dependent irradiation response of high entropy alloys. J. Alloys Compd..

[8] Sadeghilaridjani M., Muskeri S., Pole M., Mukherjee S. (2020). High-Temperature Nano-Indentation Creep of Reduced Activity High Entropy Alloys Based on 4-5-6 Elemental Palette. Entropy.

[9] Yu Y., Liu X., Wang Q.Q., Yang Q.G., Dong Y., Zhu T., Wang B.Y., Cao X.Z. (2025). Enhanced irradiation swelling resistance in low activation Ti_8_VCrMnFeCu_2_ high entropy alloys: Exploring the role of heterogeneous nanoparticles. J. Mater. Res. Technol..

[10] Amanzhulov B., Ivanov I., Uglov V., Zlotski S., Ryskulov A., Kurakhmedov A., Sapar A., Ungarbayev Y., Koloberdin M., Zdorovets M. (2024). Radiation Resistance of High-Entropy Alloys CoCrFeNi and CoCrFeMnNi, Sequentially Irradiated with Kr and He Ions. Materials.

[11] Li J., Zhu Y.X., Zhao L., Liang S., Huang M.S., Li Z.H. (2024). Interactions between edge dislocation and irradiation dislocation loop in BCC refractory high entropy alloys and the lattice distortion effect on irradiation hardening behavior. J. Alloys Compd..

[12] Zhang Q., Li L., Huang H., Chen S., Jia N., Dong Y., Guo X., Jin K., Xue Y. (2024). Radiation-enhanced precipitation and the impact on He bubble formation in V-Ti-based refractory alloys containing interstitial impurities. J. Nucl. Mater..

[13] Xu Q., Yuan X., Eckert J., Şopu D. (2024). Crack-healing mechanisms in high-entropy alloys under ion irradiation. Acta Mater..

[14] Diao S.Z., Zhao Q., Wang S.L., Han W.T., Wang Z.Q., Liu P.P., Chen Y.H., Wan F.R., Zhan Q. (2022). The microstructure evolution and irradiation hardening in 15Cr-ODS steel irradiated by helium ions. Mater. Charact..

[15] Feldmann G., Fichtner P.F.P., Zawislak F.C. (2004). Investigation of the effects of He bubbles on the nucleation, growth and thermal stability of Al–Cu nanoprecipitates in ion implanted Al foils. Acta Mater..

[16] Pu G., Lin L.W., Ang R., Zhang K., Li B., Liu B., Peng T., Liu S.F., Li Q.R. (2020). Outstanding radiation tolerance and mechanical behavior in ultra-fine nanocrystalline Al1.5CoCrFeNi high entropy alloy films under He ion irradiation. Appl. Surf. Sci..

[17] Cheng G.M., Xu W.Z., Wang Y.Q., Misra A., Zhu Y.T. (2016). Grain size effect on radiation tolerance of nanocrystalline Mo. Scr. Mater..

[18] Fradera J., Cuesta-Lopez S. (2014). Impact of nuclear irradiation on helium bubble nucleation at interfaces in liquid metals coupled to permeation through stainless steels. Fusion Eng. Des..

[19] Zhang H.Z., Wang Q.Q., Li C.H., Zhu Z.B., Huang H.F., Lu Y.P. (2023). He-ion Irradiation Effects on the Microstructures and Mechanical Properties of the Ti-Zr-Hf-V-Ta Low-Activation High-Entropy Alloys. Materials.

[20] Bauyrzhan A., Igor I., Vladimir U., Sergey Z., Azamat R., Alisher K., Maxim Z. (2023). Composition and Structure of NiCoFeCr and NiCoFeCrMn High-Entropy Alloys Irradiated by Helium Ions. Materials.

[21] El Atwani O., Vo H.T., Tunes M.A., Lee C., Alvarado A., Krienke N., Poplawsky J.D., Kohnert A.A., Gigax J., Chen W.Y. (2023). A quinary WTaCrVHf nanocrystalline refractory high-entropy alloy withholding extreme irradiation environments. Nat. Commun..

[22] El-Atwani O., Nathaniel J.E., Leff A.C., Muntifering B.R., Baldwin J.K., Hattar K., Taheri M.L. (2017). The role of grain size in He bubble formation: Implications for swelling resistance. J. Nucl. Mater..

[23] Li Z.B., Cao Y.J., Liu W., Wang Y.G., Sun F.Y., Chen Z., Zhang Z.Y. (2018). Enhanced irradiation resistance and thermal conductivity of SiC induced by the addition of carbon under Au2+ ion irradiation. Ceram. Int..

[24] Yvon P., Carre F. (2009). Structural materials challenges for advanced reactor systems. J. Nucl. Mater..

[25] Cao P.P., Wang H., He J.Y., Xu C., Jiang S.H., Du J.L., Cao X.Z., Fu E.G., Lu Z.P. (2021). Effects of nanosized precipitates on irradiation behavior of CoCrFeNi high entropy alloys. J. Alloys Compd..

[26] Wang Y., Zhang K., Feng Y., Li Y.S., Tang W.Q., Wei B.C. (2018). Evaluation of Radiation Response in CoCrFeCuNi High-Entropy Alloy. Entropy.

[27] Wang Z.J., Liu C.T., Dou P. (2017). Thermodynamics of vacancies and clusters in high-entropy alloys. Phys. Rev. Mater..

[28] Lescoat M.L., Ribis J., Gentils A., Kaïtasov O., de Carlan Y., Legris A. (2012). In situ TEM study of the stability of nano-oxides in ODS steels under ion-irradiation. J. Nucl. Mater..

[29] Watanabe H., Yamasaki K., Higashijima A., Taguma H., Nagasaka T., Muroga T. (2016). Microstructural changes of Y-doped V-4Cr-4Ti alloys after ion and neutron irradiation. Nucl. Mater. Energy.

[30] Jin Y.N., Jiang Y., Yang L.T., Lan G., Odette G.R., Yamamoto T., Shang J., Dang Y. (2014). First principles assessment of helium trapping in Y_2_TiO_5_ in nano-featured ferritic alloys. J. Appl. Phys..

[31] Hadraba H., Chlup Z., Dlouhy A., Dobes F., Roupcova P., Vilemova M., Matejicek J. (2017). Oxide dispersion strengthened CoCrFeNiMn high-entropy alloy. Mater. Sci. Eng. A.

[32] Shu S., Almirall N., Wells P.B., Yamamoto T., Odette G.R., Morgan D.D. (2018). Precipitation in Fe-Cu and Fe-Cu-Mn model alloys under irradiation: Dose rate effects. Acta Mater..

[33] Barashev A.V., Golubov S.I., Bacon D.J., Flewitt P.E.J., Lewis T.A. (2004). Copper precipitation in Fe–Cu alloys under electron and neutron irradiation. Acta Mater..

[34] Zhang S., Yao Z., Zhang Z., Oleksandr M. (2020). Irradiation damage and mechanical properties in Fe-Au and Fe-Cu model alloys under helium ion irradiation. Appl. Surf. Sci..

[35] Jin S.X., Cao X.Z., Cheng G.D., Lian X.Y., Zhu T., Zhang P., Yu R.S., Wang B.Y. (2018). Thermally promoted evolution of open-volume defects and Cu precipitates in the deformed FeCu alloys. J. Nucl. Mater..

[36] Ye F.J., Zhu T., Wang Q.Q., Song Y.M., Zhang H.Q., Zhang P., Kuang P., Yu R.S., Cao X.Z., Wang B.Y. (2022). Positron annihilation study of open volume defects and Cr segregation in deformed CoCrFeMnNi alloy. Intermetallics.

[37] Ye F.J., Zhu T., Song Y.M., Wang Q.Q., Zhang P., Kuang P., Liu F.Y., Yu R.S., Xu Q., Wang B.Y. (2021). Irradiation swelling resistance characteristics of heterogeneous nanoparticles in an iron-based multi-principal alloy. J. Alloys Compd..

[38] Zhang R.G., Wang B.Y., Zhang H., Wei L. (2005). Influence of sulfidation ambience on the properties of nanocrystalline ZnS films prepared by sulfurizing the as-sputtered ZnO films. Appl. Surf. Sci..

[39] Zhu T., Cao X.Z. (2020). Research progress of hydrogen/helium effects in metal materials by positron annihilation spectroscopy. Acta Phys. Sin..

[40] Ye F.J., Zhu T., Mori K., Xu Q., Song Y.M., Wang Q.Q., Yu R.S., Wang B.Y., Cao X.Z. (2021). Effects of dislocations and hydrogen concentration on hydrogen embrittlement of austenitic 316 stainless steels. J. Alloys Compd..

[41] Abhaya S., Rajaraman R., Kalavathi S., Amarendra G. (2015). Positron annihilation studies on FeCrCoNi high entropy alloy. J. Alloys Compd..

[42] Brusa R.S., Deng W., Karwasz G.P., Zecca A. (2002). Doppler-broadening measurements of positron annihilation with high-momentum electrons in pure elements. Nucl. Instrum. Meth. B.

[43] He S.M., Brandhoff P.N., Schut H., He S.M., Brandhoff P.N., Schut H., van der Zwaag S., van Dijk N.H. (2013). Positron annihilation study on repeated deformation/precipitation aging in Fe–Cu–B–N alloys. J. Mater. Sci..

[44] Pogrebnjak A.D. (2013). Structure and Properties of Nanostructured (Ti-Hf-Zr-V-Nb)N Coatings. J. Nanomater..

[45] Zhong Z.H., Xu Q., Mori K., Tokitani M. (2019). Thermal stability of microstructures and mechanical properties in a Fe-based Fe-Cr-Mn-Cu-Mo multi-component alloy. Philos. Mag..

[46] Liu M.W., Zheng R.X., Li H.X., Wei Q.W., Ma C.L., Tsuji N. (2024). Phase decomposition behavior and its impact on mechanical properties in bulk nanostructured Cu-20 at.% Fe supersaturated solid solution. J. Mater. Sci. Technol..

[47] Liang C., Han J.N., Zhou B.W., Xue Y.Y., Jia F., Zhang X.G. (2014). Effects of rolling and annealing on microstructures and properties of Cu–Mg–Te–Y alloy. Trans. Nonferrous Met. Soc. China.

[48] Ma Q.D., Zhang Y.B., Yue S.P., Chen J.L., Li Y.J. (2024). Microstructure and property evolution of Cu-Fe alloys during cryogenic rolling and aging treatment. Metal Heat Treat..

[49] Pogrebnjak A.D., Bakharev O.G., Pogrebnjak N.A., Tsvintarnaya Y.V., Shablja V.T., Sandrik R., Zecca A. (2000). Certain features of high-dose and intensive implantation of Al ions in iron. Phys. Lett. A.

[50] Dai H.Y., Ye F.J., Li T., Chen Z.P., Cao X.Z., Wang B.Y. (2019). Impact of Li doping on the microstructure, defects, and physical properties of CuFeO_2_ multiferroic ceramics. Ceram. Int..

[51] Nagai Y., Takadate K., Tang Z.H., Ohkubo H., Sunaga H., Takizawa H., Hasegawa M. (2003). Positron annihilation study of vacancy-solute complex evolution in Fe-based alloys. Phys. Rev. B.

[52] Xu Q., Yoshiie T., Sato K. (2008). Formation of Cu precipitates and vacancy clusters in neutron-irradiated Fe-Cu alloys. Philos. Mag. Lett..

