# Supplementary material for: Effect of Annealing on Vacancy-Type Defects and Heterogeneous Cu Precipitation Behavior in Fe60Cr12Mn8Cu15Mo3V2 Alloy
Source: Materials (Basel). 2025 Jun 3;18(11):2613. doi: 10.3390/ma18112613 (PMC12155920; doi:10.3390/ma18112613)
Supplement: Supplementary file 1 [file materials-18-02613-s001.zip › materials-3488020-supplementary.pdf]

Supplementary Materials

Effect of Annealing on Vacancy-Type Defects and Heterogeneous Cu Precipitation Behavior in $Fe_{60}Cr_{12}Mn_8Cu_{15}Mo_3V_2$ Alloy

Table S1. Positron annihilation parameters of samples at different annealing temperatures.

Anneal Temperature(K)	τ_1 (ps)	I_1 (%)	τ_2 (ps)	I_2(%)
300	91.5（±3.7）	60.5 (±3.5)	164.8(±5.4)	39.5(±3.5)
373	88.5（±3.2）	60.5(±2.9)	167.3(±4.9)	39.5(±2.9)
473	88.2（±3.1）	60.0(±2.7)	168.1(±4.5)	40.0(±2.7)
573	107.5（±1.6）	72.6(±1.9)	194.4(±3.2)	27.4 (±1.9)
673	95.6（±2.9）	68.7(±2.9)	179.2(±2.2)	31.3 (±2.9)
773	102.1（±2.8）	74.6 (±2.9)	190.3(±1.7)	25.4(±2.9)
873	96.0（±2.4）	69.1 (±2.0)	182.1(±4.2)	30.9(±2.0)
973	102.1（±0.3）	64.7 (±0.6)	179.8(±2.1)	35.3(±0.6)

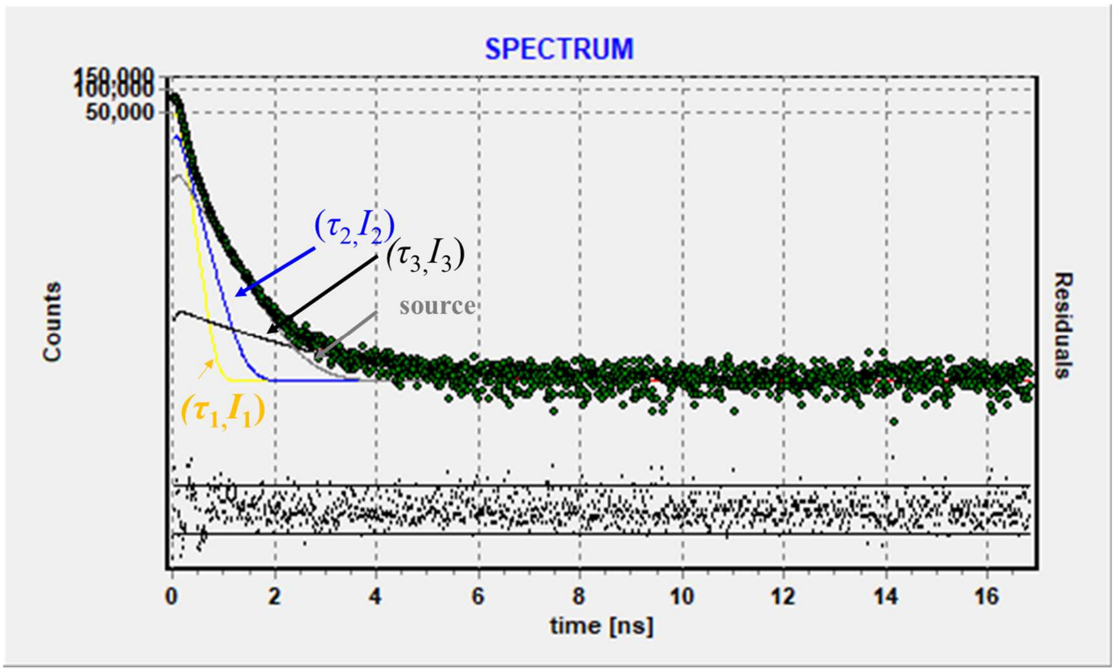

Figure S1. PAS spectra and relative fitting curves of samples annealed at 373 K.
